# Transmissible amyloid protein: evidence from iatrogenic CJD

**DOI:** 10.1007/s00415-018-8927-3

**Published:** 2018-06-13

**Authors:** Mo Hu, Neil P. Robertson

**Affiliations:** Department of Neurology, Institute of Psychological Medicine and Clinical Neurosciences, Cardiff University, Cardiff, CF14 4XW UK

## Introduction

Creutzfeldt–Jakob disease (CJD) is a progressive neurodegenerative condition caused by the accumulation of toxic forms of prion protein in the brain. In 1985, reports emerged of patients who had developed CJD following treatment with cadaveric human growth hormone contaminated with prion protein, suggesting a transmissible aetiology. More recently detailed pathological analyses of brain tissue in these patients have unexpectedly revealed the co-existence of amyloid-beta protein (Aβ), the most common misfolded protein seen in the ageing brain, which is also implicated in the aetiology of Alzheimer’s disease and cerebral amyloid angiopathy.

Both prion protein and Aβ protein share similar properties including the ability to survive normal sterilisation procedures. They are both able to grow and spread via nucleation-dependent polymerisation processes, in which oligomeric seeds can promote the formation of an ordered nucleus that can form larger fibrils. The long incubation periods of these proteins can result in clinical and pathological evidence of disease becoming apparent decades after inoculation.

In this month’s journal club, we review three recent studies that explore the potential transmissibility of Aβ pathology and which may have far-reaching implications. Two papers examine the pathological phenotype of protein accumulation in patients who died from iatrogenic CJD following growth hormone supplementation. Both suggest that the high proportion of Aβ pathology seen in this group may indicate that the Aβ protein is transmissible via contaminated human growth hormone. The authors in these papers also suggest that contamination may occur via other sources such as human dura mater grafting and neurosurgical instrumentation. The third and most recent paper attempts to shed light on the aetiology of young-onset cerebral amyloid angiopathy in patients who had undergone childhood neurosurgery.

## Evidence for human transmission of amyloid-beta pathology and cerebral amyloid angiopathy

In this study, autopsy cases of eight patients who had died from iatrogenic CJD having received human growth hormone were reviewed. Co-existence of significant Aβ pathology in four of the eight cases (aged between 36 and 51) was identified. Three of the remaining patients also showed evidence of localised Aβ pathology. There was evidence of leptomeningeal amyloid angiopathy in 3 of the patients and focal amyloid angiopathy in one patient. The authors controlled for other genetic predispositions for alternative neurodegenerative conditions including Alzheimer’s disease, amyotrophic lateral sclerosis, fronto-temporal dementia and Parkinson’s disease. Screening was performed for variations in ApoE and 16 other alleles conferring a risk for early onset neurodegenerative conditions. When compared to a cohort of other CJD patients (85 sporadic CJD, 2 variant CJD and 29 inherited prion disease) there was little or no evidence of Aβ in similar age groups using four different quantification methods. The group with similar levels of Aβ pathology was sporadic CJD patients who were of a much greater age (> 52).

As a result of these observations, the authors propose that a potential source of Aβ seeding could have been transmission via human growth hormone. The authors went on to analyse the pituitary glands of 55 patients, 49 of whom had evidence of cerebral Aβ pathology. They found that in 7 of 49 patients with parenchymal Aβ pathology, that there was also evidence of Aβ in the pituitary glands. The authors conclude that in patients with cerebral Aβ pathology, the existence of Aβ in their pituitary glands could have been a source of transmission into the human growth hormone pool.

*Comment*. This study suggests that this Aβ pathology in iatrogenic CJD patients is novel in comparison to pathology found in older patients with amyloid angiopathy. However, it remains unclear whether it is the prion protein that ‘seeds’ the Aβ protein. Other studies with larger cohorts have found significant Aβ pathology in the brains of sporadic CJD patients, raising the possibility that prion protein induces endogenous Aβ production. Against this theory is the authors’ observations that the prion protein and Aβ were not co-localised in their specimen samples. Despite the presence of Aβ, none of the patients exhibited clinical features of cerebral amyloid angiopathy or Alzheimer’s disease before death. Furthermore, no patients exhibited histological evidence of other proteins implicated in the latter stages of Alzheimer’s disease pathology, such as tau protein.

The study is exploratory in nature and uses small numbers of cases which were analysed retrospectively from one centre. It is, therefore, difficult to draw definite conclusions about causality. Larger scale studies are needed to replicate these results, and experiments designed to explore whether or not Aβ protein is ‘seeded’ by prion protein.

Jaunmuktane Z et al (2018) Acta Neuropathol 135(5):671–679.

## Amyloid-beta accumulation in the CNS in human growth hormone recipients in the UK

This study investigated a large cohort of human growth hormone recipient cases together with novel controls exploring the presence of prion protein and Aβ pathology. The authors analysed the brains of 35 patients who died from iatrogenic CJD following growth hormone supplementation, as well as five who developed CJD following human dura mater transplantation. They also utilised a group of 12 age-matched controls who had received human growth hormone, but who did not go on to develop CJD. The authors sought to characterise the spread and distribution of prion protein according to genotypic influences, whilst again controlling for genetic predispositions for neurodegenerative diseases.

The authors report that polymorphisms of endogenous prion protein at codon 129 did seem to determine the distribution of prion protein, similar to that in sporadic CJD, and also influence disease duration, with MV heterozygosity of codon 129 conveying the longest disease duration. Further analysis revealed evidence of prion protein in non-CNS tissues, including nerves, dorsal root ganglia and trigeminal ganglia in small numbers of non-CNS samples.

Aβ pathology was detected in 18 out of the 33 growth hormone patients who had developed iatrogenic CJD. This was seen as parenchymal deposits or cerebral amyloid angiopathy (6 amyloid angiopathy, 4 parenchymal Aβ, 8 both). Importantly, 5/12 human growth hormone controls (who did not develop iatrogenic CJD) also showed CNS Aβ accumulation (three parenchymal, one limited amyloid angiopathy, one diffuse amyloid angiopathy and cortical plaques). In neither group was the accumulation of Aβ sufficient to make a pathological diagnosis of Alzheimer’s disease, nor was there clinical evidence of Alzheimer’s disease. Similar proportions of patients exhibiting Aβ pathology were not seen in 33 variant CJD patients nor the 15 (older) sporadic CJD patients. There was also little evidence of co-localisation of prion protein and Aβ, further suggesting that prion protein could not be the lone precipitant in ‘seeding’ endogenous Aβ pathology. None of the human dura mater transplantation patients had evidence of Aβ pathology.

The presence of Aβ in patients with iatrogenic CJD did not correlate with their length of growth hormone treatment nor their age at death, however, in human growth hormone recipients who did not develop iatrogenic CJD, being positive for Aβ was moderately correlated with earlier and longer duration of treatment with human growth hormone. There was no significant correlation between APOE-e3/4 or APOE-4 positivity and Aβ pathology and there was no significant influence of genetic predispositions for neurodegenerative diseases.

The authors conclude that Aβ pathology could be transmitted via growth hormone supplementation independently of prion protein.

*Comment*. This study uses large numbers of patients, and advanced techniques in detection and stratification of both prion protein and Aβ. Genotypic analysis of the prion protein at codon 129 gives us some clues as to the genotypic influences which determine where the contaminated prion protein is located, and the likely disease severity measured according to disease duration before death. Given the small numbers of non-CNS samples analysed it is difficult to suggest peripheral rather than CNS transmission of prion protein.

Importantly, the similar levels of Aβ in the human growth hormone recipient group who did not develop iatrogenic CJD points to independent transmission of both proteins. Care needs to be taken in analysing this group because of small numbers (12) and by the presence of possible other co-morbidities causing their significantly shortened life span.

Aβ pathology accumulation seems to be a dynamic process, and the use of purely autopsy date does limit our interpretations as to the growth and spread of Aβ pathology with time. Significantly, in this study, none of the patients exhibited the clinical features of a neurodegenerative condition despite the presence of Aβ pathology. This may be in part due to the fact that patients who developed Aβ following growth hormone supplementation died at a young age.

Ritchie DL et al (2017) Acta Neuropathol 134(2):221–240.

## Evidence of amyloid-β cerebral amyloid angiopathy transmission through neurosurgery

This study comprises a retrospective analysis of a case series of young-onset cerebral amyloid angiopathy presenting in to a UK centre between 2002 and 2016. The authors identified five biopsy-proven cases and one-autopsy proven case in a pathology archive, and six cases from the published literature. None of these patients had evidence of CJD. The authors interrogated case records for evidence of early childhood neurosurgical procedures as a predisposing factor for cerebral amyloid angiopathy.

In 3/5 of the biopsy cases, detailed history taking confirmed childhood neurosurgical intervention (secondary to trauma, congenital malformations and tumour resection). All of these patients had presented to hospital decades later following an intracerebral haemorrhage, and had their biopsy taken at that point. In the single autopsy case from a patient who had died following intracerebral haemorrhage, there was also a history of early neurosurgical intervention (secondary to syringomyelia). In all of these pathological cases, there was histological evidence of diffuse Aβ pathology. When samples of 50 age-matched controls (who had undergone cerebral biopsy for other reasons) were analysed, no evidence of Aβ pathology was identified.

A supplementary literature review of early onset cerebral amyloid angiopathy patients identified six cases. In all cases there was evidence of previous penetrating brain injury. In two cases there was also a record of childhood neurosurgical intervention, whilst a further two had radiological evidence of early-life neurosurgical intervention. Since there was no history of penetrating traumatic brain injury in their own pathological cohort, the authors conclude that the most likely correlate of young-onset cerebral amyloid angiopathy appears to be early-life neurosurgical intervention. Furthermore, the authors suggest that Aβ proteopathic seeds may have been transmitted by surgical instruments carrying traces of misfolded Aβ.

*Comment*. This study is the first to draw a direct link between a possible route of Aβ transmission and a clinical manifestation of Aβ pathology. The study used focussed clinical history to determine pre-existing factors that may have predisposed to the development of cerebral amyloid angiopathy.

The study did draw from biopsy samples taken in vivo, thus not all data were retrospective. However, without prospective modelling it is difficult to adequately characterise the clinical phenotypes of these patients. Indeed there is limited clinical information for a number of these patients, including the details of relevant neurosurgical procedures. Furthermore, the evidence of early penetrating traumatic brain injury in the literature review group cannot be discounted as a potential cause of Aβ accumulation.

Although a high incidence of Aβ was seen in this cohort of patients, care needs to be taken in extrapolating from such a small sample. Cerebral amyloid angiopathy patients above 55 were excluded in the biopsy cohort, and those over 57 were excluded in the autopsy group. Perhaps including older patients in their analysis would increase numbers and provide further information about predisposing factors for development of cerebral amyloid angiopathy, although the existence of ‘sporadic’ Aβ protein in this age group would be a confounding problem. Further studies looking prospectively at the development of Aβ pathology in well-characterised controlled groups of patients undergoing neurosurgery may be of benefit.

Jaunmuktane Z et al (2018) Acta Neuropathol 135(5):671–679.

*Conclusions*. These studies highlight a rare but important possible sequelae of growth hormone supplementation, as well as other possible sources of protein seeding, including surgical instrumentation. The studies have all used up to date- and highly advanced techniques in characterising the presence of Aβ as well as prion protein, and in controlling for other co-morbidities. Collectively, these studies present important preliminary evidence for the transmissibility of Aβ protein in humans. The authors’ hypotheses are also supported by evidence of iatrogenic transmissibility of Aβ in murine models by both intracerebral and peripheral routes.

All patients who developed iatrogenic CJD had growth hormone via the Wilhelmi preparation, and there is a suggestion that techniques used in this preparation specifically allow prion protein as well as Aβ to survive within the growth hormone batch. There is less evidence for the presence of Aβ protein being present on neurosurgical instruments, and this could be an important future focus for research. In addition, a more detailed understanding of the properties of Aβ protein that allow it to become transmissible and able to survive standard sterilisation techniques may help prevent future cases of transmission.

Future studies looking at the epidemiological clinical correlates of Aβ transmission would be of value. Studies exploring the prevalence of neurodegenerative diseases in a large population of those who have undergone human growth hormone supplementation may be relevant. Large-scale studies following up patients who have undergone brain instrumentation via neurosurgery may also be interesting. All of these studies are limited by small numbers, lack of robust clinical correlations, and difficulty extrapolating across different centres. Given the shift in modern neurosurgical techniques, sterilisation procedures, and the discontinuation of human growth hormone, it may be that the rates of transmissibility are currently much lower or absent.

